# The push-out bond strength of three root canal materials used in primary teeth: *in vitro* study

**DOI:** 10.3389/fdmed.2023.1140794

**Published:** 2023-03-24

**Authors:** Hazal Özer, Merve Abaklı İnci, Sevcihan Açar Tuzluca

**Affiliations:** Department of Pediatric Dentistry, Faculty of Dentistry, Necmettin Erbakan University, Konya, Turkey

**Keywords:** MTA (mineral trioxide aggregate), calcium hydroxide, primary tooth, root canal filling material, pediatric dentistry

## Abstract

The study aims to compare the bond strength of three primary tooth root canal filling materials to the root canal wall with a push-out test (Calplus, DiaPaste, BIOfactor MTA). First, 30 primary central teeth were cut transversely using a water-cooled low-speed diamond saw vertical to the long axis to obtain 2 mm thick discs from the middle third of the roots. Next the materials used were placed on dentin discs and kept in an incubator for 1 week at 37°C and 100% humidity until the hardening mechanism of the root-canal sealer was completed. Finally, a vertical force was placed on each material from apical to coronal with a 0.75 mm diameter stainless steel cylindrical piston without contacting the root canal dentin. The data were analyzed using the SPSS 22.0 program and the Mann-Whitney U test was used as a *post hoc* test. There was a statistically significant difference between the bonding values of different primary tooth root canal sealers to root canal dentin (*p* < 0.05). Among the maximum binding values, the lowest measurement was in Calplus (0.43 ± 0.28 MPa), and the highest measurement was in BIOfactor MTA (24.24 ± 17.78 MPa) (*p* < 0.05). BIOfactor MTA has a higher bonding value to root canal dentin than calcium hydroxide-based primary tooth canal sealers.

## 1. Introduction

One of pediatric dentistry's most critical tasks is keeping the primary teeth healthy and functional in the mouth until they exfoliate naturally. Premature loss of primary teeth may result in loss of dental arch length, insufficient space for permanent teeth to erupt, the ectopic eruption of premolars, mesial movement in the molar tooth adjacent to the extraction cavity, elongation of the opposing permanent tooth, deviation in the midline with the formation of cross occlusion, malocclusions, aesthetic, chewing, and phonation problems (1–6).

In addition, caries lesions progress faster and significantly impact the pulp-dentinal complex because of their smaller thickness and wider pulp chambers. Untreated primary tooth decay spreads quickly as well, causing loss of substance and increasing the need for root canal treatment in profound caries (7–9). The purpose of root canal treatment in primary teeth is to allow teeth that function during an essential period of life to function painlessly without damaging the underlying permanent tooth germ, to heal pathologies in the furcation and periapical region, and to allow the tooth to resorption physiologically (10).

The properties of root canal sealers used in primary tooth root canal treatment are critical. For example, an ideal primary tooth root-canal sealer should be antibacterial, resorb in parallel with primary tooth resorption in the presence of permanent teeth, not harm the periapical tissues and permanent tooth germ, and be easily resorbed when overflowing from the apex. Furthermore, ease of application, good adaptation to the canal wall, ease of removal from canals when necessary, radiopaque properties, and not causing tooth discoloration are all essential criteria (11, 12).

Although a wide range of sealers has been used in primary tooth root canal treatments, no root-canal treatment material possesses all these properties simultaneously. Hermann's introduction of calcium hydroxide paste in 1930 was indicated for use in primary teeth due to its anti-bacterial, resorbable, and biocompatible properties (13). Iodoform paste, on the other hand, demonstrated 84–100 percent success in terms of resorbing excess material and healing properties (14). The root-canal treatment materials containing a calcium hydroxide-iodoform mixture are close to ideal for primary teeth. The adaptability to the root-canal surface and the sealing properties are suitable (10).

In 1993, MTA was developed by Dr. Torabinejad; it is widely used in root canal repair, vital pulp treatments, and apical barrier formation due to its high pH, histological and biological properties similar to calcium hydroxide, excellent biocompatibility, low solubility, high sealing ability, and radiopacity (15, 16). In the studies, successful covering properties were obtained; this is attributed to the chemical properties of the material and its expansion during setting (16). Root-canal treatment materials containing MTA have been developed in recent years by improving these favorable properties of MTA and adding features such as fluidity, setting time, and adhesion that are essential in root canal sealers; it has found widespread use in endodontic treatments (17).

MTA stimulates the formation of hard tissues such as bone, dentin and cement and has a regenerative property on periapical tissues. Therefore, it has osteoconductive, osteoinductive and cementogenic properties. It stimulates the release of the lymphokines, bone-binding factors required for the repair, regeneration and bone defects of damaged cement tissue from immune cells (18).

For treatments like pulp capping, pulpotomies, apexification, root perforation repairs, root-end sealer, and apical plugs, the BIOfactor MTA employed in this study (Imicryl Dental, Konya, Turkey) has just recently entered the market. This material can be formed with a fluid or dense consistency, depending on the type of treatment. The producer of BIOfactor MTA claims that it doesn't stain teeth and has finer particle content powder for quicker hydration, easier handling, stronger sealing, and shorter setting times. The bond strength of BIOfactor MTA, a novel material, has never been examined on primary teeth. The BIOfactor MTA is also less expensive.

In a study conducted in this context, the Ph, solubility, contact angle, and crystallline microstructure under SEM and antibacterial activity were evaluated for three root-canal filling materials for primary teeth (Calplus, Bio-C Pulpecto, and zinc oxide eugenol). None of the materials had optimal properties and could be considered the most suitable filling material for primary teeth pulpectomy. However, the properties of bioceramics, such as bioactivity, solubility in fluids, and adhesiveness, would provide a crucial step in increasing the success rate of root canal treatment on primary teeth and developing more performant materials (19).

A root-canal sealer ensures the integrity of the material-dentin connection with an adhesive bond. This adhesive joint must be strong enough to withstand sealant displacement during function and operating procedures (20). This force is measured and evaluated using the push-out test. The materials to be tested are placed in cavities of a specific diameter prepared in the middle of dentin discs of a certain thickness obtained, and then the root canal sealer is pushed from the root canal with the help of a pusher tip and gives the maximum force bond strength value that allows the rupture to occur. The push-out test can also be used to assess root-canal sealing materials with low bond strength (21).

It is critical to achieve a successful root canal treatment using biocompatible, non-toxic, highly impermeable materials and suitable adaption to the canal surface in primary tooth root canal treatments. Root-canal treatment materials must have good adaptability to the root dentin surfaces. Bond strength tests are used to assess the effectiveness of endodontic material adhesion to the tooth structure. The push-out test is one of the methods used to determine the bond strength of intracanal restorations, and it more accurately models clinical conditions than other methods.

In the literature, our research is the first to investigate the bond strength of conventional primary tooth root-canal filling materials with MTA, one of the most preferred biomaterials in root canal treatments in primary teeth with no permanent. Given that different root canal sealers have different properties in bond strength to root dentin, this *in vitro* study aimed to investigate the hypothesis that the push-out test values may differ depending on the material used.

The following evidence supports this hypothesis:

Among root canal sealers, BIOfactor MTA has the highest bonding strength values to primary tooth root canal dentin.

## 2. Materials and methods

The Non-Pharmaceutical and Non-Medical Device Research Ethics Committee at Necmettin Erbakan University Dentistry gave authorization with decision number 2020/02-08 on the date of 05.11.2020.

### 2.1. Sample size calculation

From a previous study (30), sample size, effect size = 0.30, power b = 95%, *α* = 5% were calculated based on input into an F-test family for the analysis of variance repeated measurements, and for this study, 27 samples were required. However, 60 samples obtained from 30 teeth have been included to cover any potential early problems.

### 2.2. Sample preparation

In our study, we used 30 freshly extracted primary central human teeth that did not exfoliate, although they were due. Instead, the teeth were kept in tap water containing 0.1% thymol at 4 °C after extraction until used in the study. A scaler was used to remove tissue residues from the root surface. Preoperative radiographs were taken to confirm the presence of a single root canal and to confirm that the root curvature was less than 20°.

The crowns were removed after the teeth were cut from the cemento-enamel junction, perpendicular to the long axis of the tooth with a low-speed IsoMet diamond saw under constant water-cooling (IsoMet 1000; Buehler, Lake Bluff, NY, USA). The length of the roots was standardized at 8 mm. Canal sealer opening of the teeth was measured under magnification (Zumax SLT Loupe 3.0x) by exiting 0.5 mm from the apical with an ISO #10 K-type endodontic hand file (Dentsply, Maillefer, Ballaigues, Switzerland). Root canals were shaped using a protaper universal file system (Dentsply, Maillefer, Ballaigues, Switzerland) in working length up to the #30 file (%0.4 taper) and the crown down technique, as recommended by the manufacturer. The canals were irrigated with 2 ml of 5% NaOCl (Imicryl, Konya, Turkey) solution at each file type and size change. It was then washed with 2 ml of saline solution. Following the completion of the preparation process, 5 ml of 17% EDTA (Imicryl, Konya, Turkey) solution was used to remove the smear layer, which was then washed with 2 ml of saline solution. For final irrigation, 5 ml of distilled water was used. The root canals were then dried with paper cones.

The prepared teeth were embedded in cold acrylic using cylindrical molds with a diameter of 10 mm and a height of 20 mm. To obtain 2 mm thick discs in the middle third of the roots, the teeth were cut transversely with a water-cooled low-speed ISOMET diamond drill (IsoMet 1000; Buehler, Lake Bluff, NY, USA) perpendicular to the long axis of the teeth. The thickness of the resulting discs was measured using a digital caliper. The resulting discs were enlarged to 1.3 mm in diameter using Gates Glidden drills number 2, 3, and 4, respectively (Dentsply, Maillefer, USA). All discs were washed with 5 ml of distilled water and dried with paper cones afterward.

Sixty dentin discs were randomly divided into three groups (*n* = 20). Root-canal sealers (BIOfactor MTA, Calplus, and DiaPaste) were placed in the root canal cavity of the discs with the carrier and compressed with an endodontic plugger according to the manufacturer's instructions. (Table 1) A scalpel was used to remove excess material from the samples' surfaces. For one week, all discs were kept in an oven at 37°C and 100% humidity until the setting mechanism of the root-canal sealers was completed.

**Table 1:** Root-canal sealers used in the study, as well as the content and manufacturer information. .

Product and manufacturer	Composition	Instructions for use
BIOfactor MTA (Imicryl Dental, Konya, Turkey).	Powder: tricalcium silicate, dicalcium silicate, tricalcium aluminate, ytterbium oxide as radioactive softener	Mix 3 scoops of powder with 1 drop of liquid until you get a homogeneous consistency
	Liquid: 0.5%–3% water-soluble carboxylated polymer, demineralized water	
DiaPaste (DiaDent Europe B.V.Antennestaat, the Netherlands)	Barium sulphate with pre-mixed calcium hydroxide	Inject into the canal with the syringe
Calplus (Prevest DenPro Limited EPIP Bari Brahmana, Jammu-181133, India).	Calcium hydroxide, iodoform, and silicone oil	Inject into the canal with the syringe

**Figure 1:**
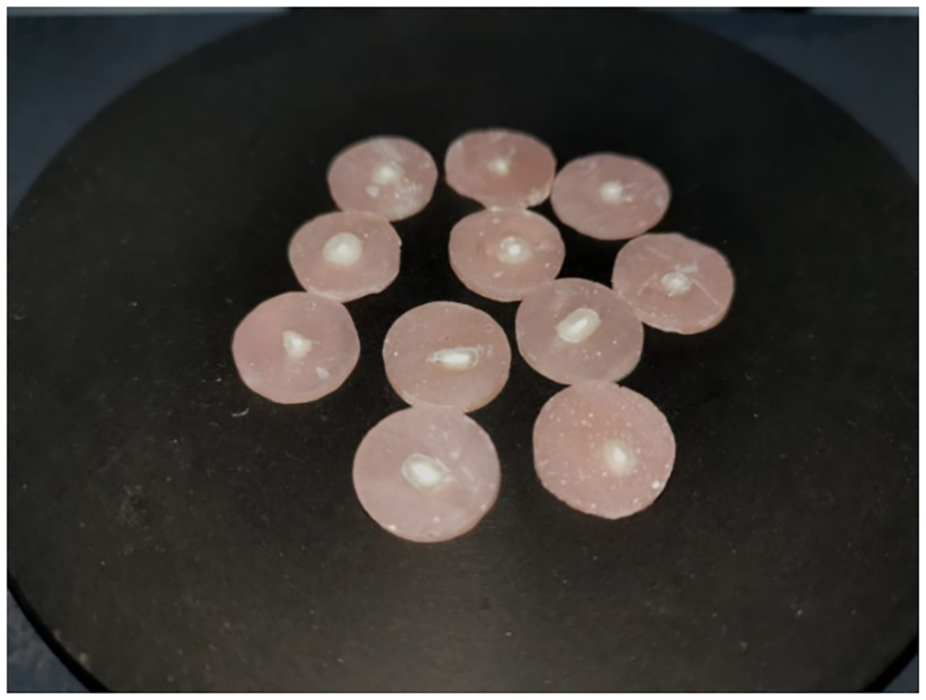
Dentin discs with root canal path before applying force.

**Figure 2:**
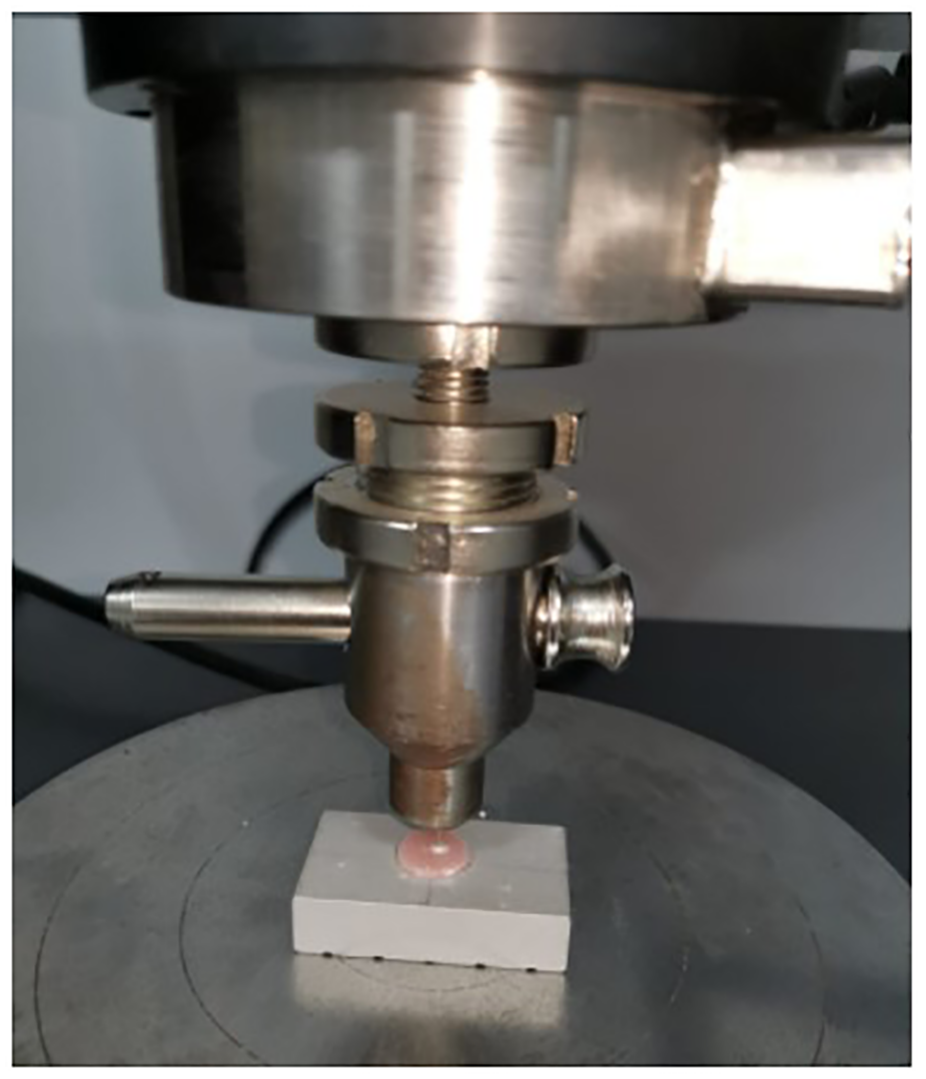
Universal testing machine.

After the sealers' setting mechanisms were completed, root surfaces were sanded to achieve a smooth and clear surface (Figure 1). Next, all discs were examined with a microscope (Olympus Optical Co. Ltd., Tokyo, Japan) to look for cracks, imperfections, or gaps between the sealer and the dentinal walls. It was then put through a push-out test on the Universal Testing Machine (Universal, Beyhekim, Turkey) (Figure 2).

### 2.3. Push-out testing

**Figure 3:**
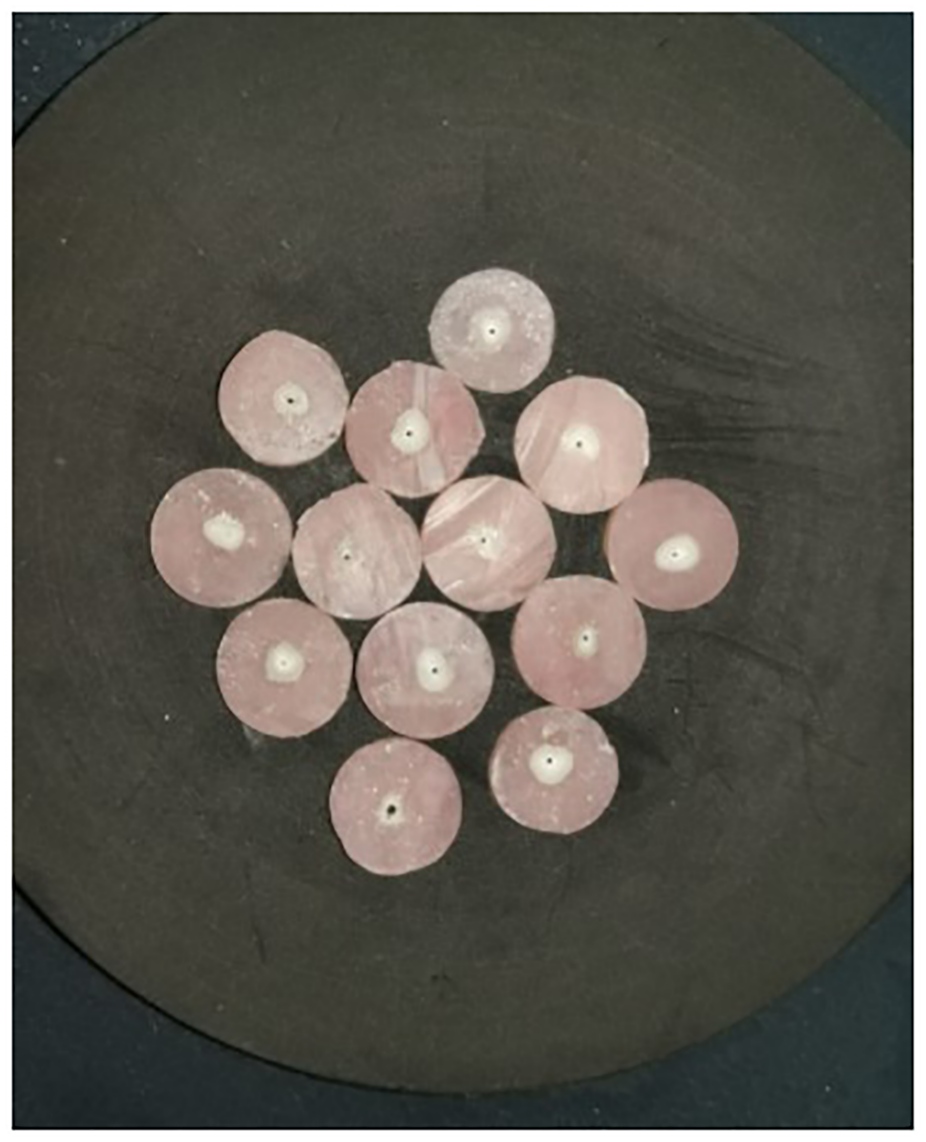
Dentin discs after force is applied.

The discs were placed in a steel holder that was screwed to an alignment device centered beneath a cylindrical steel punch. After that, the alignment device was attached to the universal tester, Instron machine (Model 4444; Instron Corp, Canton, MA). The thruster had a 0.5 mm tip, and the thrust rate was set to 1 mm/min. In each sample, a vertical force was applied to the cement. Then, using a 0.75 mm diameter stainless steel cylindrical piston, the force was applied to the sealing material from apical to coronal, providing the most coverage on the sealing material without coming into contact with the surrounding dentin (Figure 3).

The maximum force exerted on the cement before displacement was measured in Newtons (N). Thrust force was calculated in megapascals (Mpa) by dividing the force (N) by the area in mm^2^. The maximum load needed to cause a sealing failure was measured in Newtons. The obtained data were converted to (Mpa) using the formula (where is π constant and shows canal radius and root slice thickness every two millimeters (newton/2πrh).

### 2.4. Failure mode analysis

Following the push-out test, samples were inspected at a 500× magnification under a stereomicroscope (SZTP; Olympus Optical Co., Tokyo, Japan) to assess the likelihood of mixed, cohesive, or adhesive failure at the dentin-material interface.

### 2.5. Scanning electron microscopy

**Figure 4:**
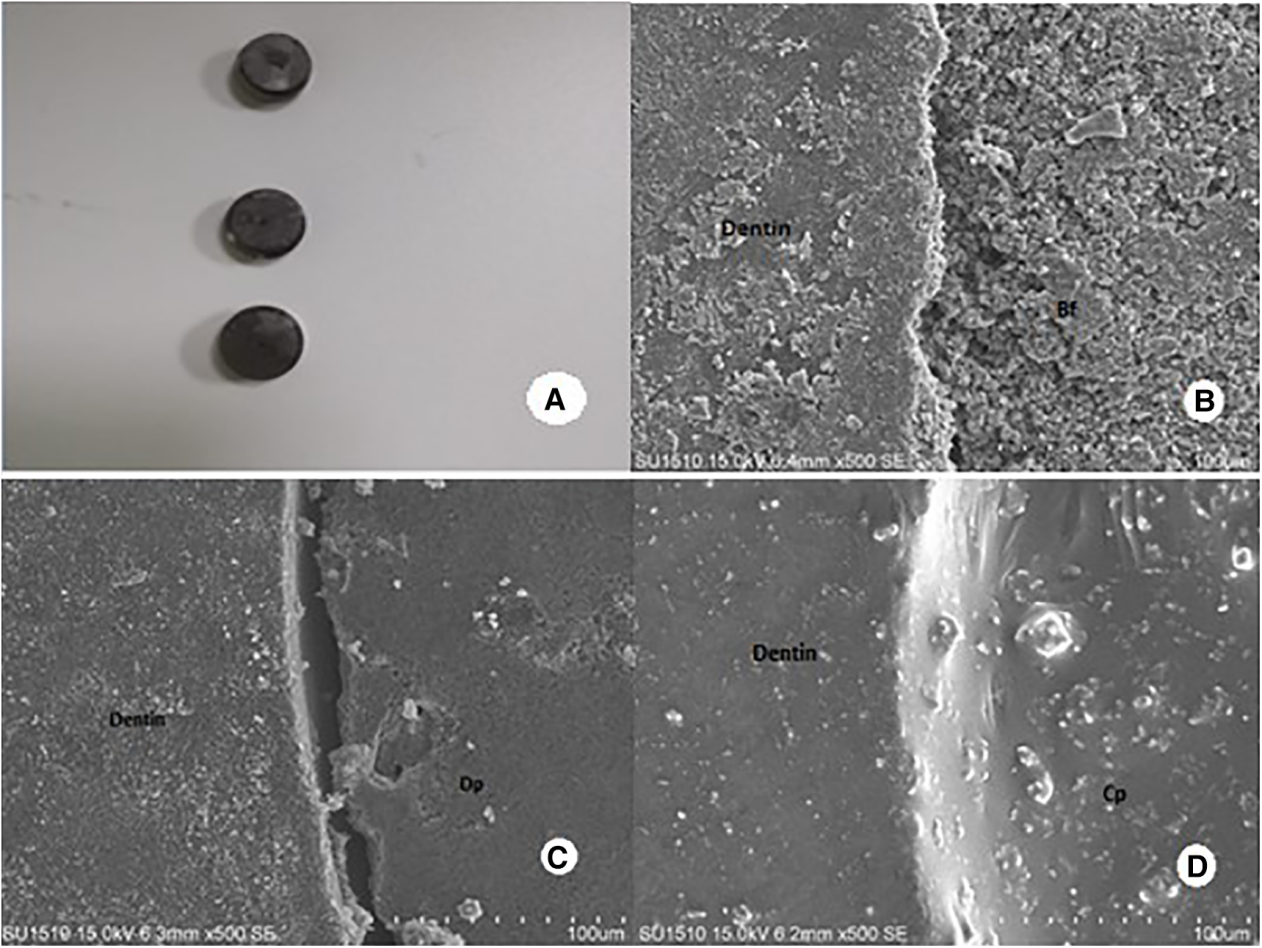
SEM images. (**A**) Dentin discs coated with Ag-Pd are randomly selected. (**B–D**) Cohesive failure modes in representative scanning electron micrographs of the root canal sealer-dentin interface after push-out test. (**B**) BIOfactor MTA (Bf), (**C**) DiaPaste, and (**D**) Calplus (original magnification 500×).

Six dentin slices (two from each group) were chosen for scanning electron microscopy (SEM) analysis. For 3 min, samples subjected to the push-out test were coated with gold/palladium. The interface between dentin and root repair material was explicitly observed in samples. Scanning electron microscopy (Hitachi SU-1510; Hitachi High-Technologies Corp., Tokyo, Japan) was used to make observations at a magnification of 500× (Figure 4).

### 2.6. Statistical investigation

The Shapiro-Wilk test indicated that the data were normally distributed. The Kruskal-Wallis test was used for intergroup comparisons within the scope of the study, and the Mann-Whitney U test was used as a *post hoc* test. The data were analyzed using the SPSS 22.0 (IBM-SPSS Inc., Chicago, IL, USA) package program, and the study was conducted with *p* < 0.05 as a reference at the 95% confidence interval.

## 3. Results

Table 2 displays the mean, standard deviation, lowest, and highest bond strength values obtained from the study's groups. The maximum force measurements differ between the three groups statistically significantly. Calplus has the lowest, and BIOfactor MTA has the highest measure. The Mann-Whitney U test for pairwise comparisons revealed no statistically significant difference between DiaPaste and Calplus. However, maximum force measurements between DiaPaste and BIOfactor MTA revealed a statistically significant difference, with the BIOfactor MTA measurement being higher. Maximum force measurements between BIOfactor MTA and Calplus show a statistically significant difference, and it was determined that the BIOfactor MTA measurement was higher.

**Table 2:** Push-out bond strength values [Mpa].

DiaPaste	BIOfactor MTA	Calplus	*p*
1,37 ± 1,32 (0,43–4,73)^a^	24,24 ± 17,78 (3,43–81,15)^b^	0,43 ± 0,28 (0,13–3,76)^a^	0,001

med ± ss (min-max)-.

^a,b^ When comparing groups, marks are used to indicate significant differences.

The failure mode analysis results are represented in Table 3. Instead of mixed failures, all of the groups displayed coherent failure majorities under the stereomicroscope.

**Table 3:** Failure mode results (%).

Failure type	Calplus	DiaPaste	BIOfactor MTA
Adhesive	0 (0)	1 (1.66)	0 (0)
Cohesive	57 (95.0)	58 (96.67)	58 (96.67)
Mixed	3 (5)	1 (1.66)	2 (3.33)

## 4. Discussion

This study aims to use the push-out test to compare the bond strength of three different primary tooth root canal sealer materials on the root canal surface. This is the first study in the literature to investigate the bond strength of DiaPaste, Calplus, and BIOfactor MTA root canal sealers to root dentin. The hypothesis was confirmed when a statistically significant difference in push-out test bond strength was discovered between root canal sealers used.

The primary goal of root canal treatment is to clean, shape, and seal the root canal in three dimensions. The connection of root canal sealer material with dentin is directly related to the sealing of the root canal sealer (22). As root canal sealers' bonding ability to the root canal surface improves, so does the success rate of endodontic treatment (23).

Different test methods, such as the widely used push-out test and the traditional shear test, can be used to evaluate the adhesion of root canal sealers to the root-canal surface (24). Bond strength is also measured using tensile tests. It determines bond strength by pulling the canal sealer applied to the dentin discs with a tip. According to the studies, a wide range of values was obtained in the tension tests, and as a result, the push-out test method was more reliable (25). The push-out test has been reported to be a reliable and practical test for evaluating its adhesion to root dentin. Furthermore, it has many advantages, such as more closely stimulating clinical stress, allowing accurate disc standardization, being effective, reliable, and practical, and producing purer shear forces (26). As a result, the push-out test was chosen for this study.

The bond strength of various intra-root posts is affected by the type of root canal sealer material used. The binding values of zinc oxide eugenol were weaker than Metapex, a primary teeth canal sealer based on iodoform and calcium hydroxide, in a study comparing the bonding strength of three types of intracanal posts using the push-out test (27). Another study with primary anterior teeth found that the bond strength values of root canal posts treated with Metapex were more remarkable than those of zinc oxide eugenol (28).

The results of Machida et al. show that the calcium hydroxide-iodoform mixture satisfies the requirements for an optimal primary tooth canal sealing material (29). Vitapex (Neo Dental, Tokyo, Japan) and Metapex (Meta Biomed, Cheongju, Korea) are canal-sealing materials with strong antiseptic properties designed for primary teeth. It is simple to apply/remove root canals. In primary tooth pulpectomy, Vitapex or Metapex has been associated with significant clinical and radiological success rates (30).

The use of root canal sealers containing iodoform or calcium hydroxide instead of zinc oxide eugenol has increased dramatically in recent years (31). Calplus and DiaPaste were preferred as calcium hydroxide-based root canal sealers in our study, while BIOfactor MTA was chosen as a silicate-based root canal sealer. In an *in vitro* study examining the bond strength of BIOfactor MTA with the push-out test, BIOfactor MTA exhibited high bond strength to root canal dentin, at least as much as MTA-Angelus and Biodentine (32).

There was no statistically significant difference between ProRoot and BIOfactor MTA in clinical and radiological examination in the first 6 months of a clinical study evaluating the long-term success of BIOfactor MTA and ProRoot MTA in vital pulpotomy in primary molar teeth. However, at a 12-month follow-up, ProRoot MTA statistics were found to have a significantly higher clinical and radiological success rate than BIOfactor MTA (33). More research is needed to determine whether the ytterbium oxide substitution in BIOfactor MTA powder affects its chemical bonding to dentin.

The BIOfactor MTA was enhanced with ytterbium oxide as a radiopacifier substance, in contrast to other calcium silicate-based materials. It is unclear how the ytterbium oxide addition to the calcium silicate-based substance will affect its physicochemical characteristics. However, ytterbium trifluoride has been added to calcium silicate-based materials. It was found that doing so improved the material's porosity while only slightly increasing the compressive strength of portland cement (34).

There was no statistically significant difference in the push-out values of the materials in a study in which the binding strengths of the ProRoot MTA, Angelus MTA, and Biodentin materials were tested. However, there was a difference between the structural performances of the materials, i.e., the types of failure. While adhesive failure in the biodentin group is never seen, Koheziv and mixed failures were seen equally. In Proot MTA and MTA Angelus groups, most of them were seen from all types of failures, and in both groups, the types of failure were seen in an equal number (35).

The findings of this study, however, are inconclusive, and additional, well-designed research is still required to fully grasp the ideal filling substances' qualifications. Calplus and DiaPaste showed comparable push-out test resistances. Compared to Calplus and DiaPaste, BIOfactor MTA has a higher binding value. BIOfactor MTA has a sufficient bonding strength to the root dentin; however, there is still room for improvement in the MTAs' attributes. Clinical investigations on the therapeutic impact and root canal bonding capability are necessary to evaluate calcium hydroxide-based DiaPaste and Calplus thoroughly. Moreover, randomized long-term clinical studies are required to evaluate the clinical behavior of this kind of material because primary molars continuously experience root resorption. The physicochemical and antibacterial properties still need to be improved to suit the complex anatomy of primary teeth.

## Data Availability

The original contributions presented in the study are included in the article/Supplementary Material, further inquiries can be directed to the corresponding author/s.
